# Using the problem based learning method and educational technologies to teach open data: A design-based research approach

**DOI:** 10.1007/s10639-022-10995-9

**Published:** 2022-03-21

**Authors:** Eleni Dermentzi, Maria Zotou, Efthimios Tambouris, Konstantinos Tarabanis

**Affiliations:** 1 Newcastle Business School, Northumbria University, City Campus East, Newcastle Upon Tyne, NE1 8ST UK; 2 Information Systems Lab, University of Macedonia, 156 Egnatia Street, GR-546 36 Thessaloniki, Greece; 3 Department of Applied Informatics, University of Macedonia, 156 Egnatia Street, GR-546 36 Thessaloniki, Greece; 4 Department of Business Administration, University of Macedonia, 156 Egnatia Street, GR-546 36 Thessaloniki, Greece

**Keywords:** Problem-based learning, Open data, Design based research, Educational technologies, Higher education

## Abstract

With Open Data becoming more popular and more public bodies publishing their datasets, the need for educating prospective graduates on how they can use them has become prominent. This study examines the use of the Problem Based Learning (PBL) method and educational technologies to support the development of Open Data skills in university students. The study follows a Design Based Research approach and consists of three phases: a) examination of stakeholders’ needs, b) design of an Open Data module, and c) re-design of the module based on the outcomes of its first run. The data collected throughout the three phases come from various sources, namely interviews with practitioners, focus groups with students, and tutors’ reflection. The findings suggest that while the PBL method is suitable for Open Data education, special care should be taken to ensure that the potential of educational technologies is fully realised. The study concludes with design principles that aim to guide instructors on how they can incorporate the PBL method and digital tools into Open Data education effectively.

**Supplementary Information:**

The online version contains supplementary material available at 10.1007/s10639-022-10995-9.

## Introduction

The availability of governmental and other information to the public promises significant added value and potential economic growth (Lassinantti et al., 2019). The main aim of opening data is to allow its exploitation and re-use in ways that they can produce unforeseen services, products and start-ups that address existing economic and societal problems. For example, data regarding the COVID-19 cases worldwide are available on European Union’s portal (European Centre for Disease Prevention and Control, 2020), which can be used to develop applications that will help the prevention and control of the disease. Another significant benefit of Open Data (OD) is to allow public transparency, encourage citizens’ engagement in policy making and improve public service delivery (Lassinantti et al., 2019; World Wide Web Foundation, 2018).

However, publishing and re-using OD requires a specific set of knowledge and skills set that are still lacking in the market, allowing only a limited percentage of OD experts to exploit OD (Weerakkody et al., 2017; World Wide Web Foundation, 2018). Existing efforts on OD education usually involve short workshops or a series of slides that present the basic concepts and do not allow practical experimentation or skills development. This calls for new opportunities in educating prospective graduates on OD, so that they acquire knowledge on a wide variety of relevant topics and gain skills that will allow them to properly work with OD on any subject field as soon as they join the workforce.

The challenge here is twofold: Firstly, the definition of the aforementioned OD skills is vague. So far, scholars have discussed the generic data literacy skills needed (e.g. data management, visualisation, etc.) (Eckartz et al., 2016; Yoon & Copeland, 2019), however, a curriculum needs to include specific learning goals regarding the OD skills and knowledge. Also, the context of the training programme needs to be taken into consideration (Fotopoulou, 2020), as a curriculum designed for a specific organisation may not be relevant to university students. Thus, there is need for developing a curriculum with specific learning goals that are appropriate for university students.

Secondly, it is unclear how university students can develop OD skills. Due to the nature of the skills, it is evident that appropriate technologies (e.g. Tableau) should be used along with the course’s Learning Management System (LMS). In addition, literature suggests that a real-life problem-solving approach should be followed by communities of practice for the potential of OD to be realised (Garwood & Poole, 2019; Susha et al., 2015). This indicates that a pedagogical method such as the Problem Based Learning (PBL) method that focuses on learning by solving problems could be potentially effective in developing OD skills. However, it is unclear whether learners will actually find the PBL method helpful for studying OD and whether educational technologies can help them follow the PBL steps in the OD context.

Based on the above, the study aims to answer the following research questions:

- What OD skills are appropriate for university students?
- What are university students’ perceptions of the PBL method as a way of developing OD skills?
- How do university students view the role of educational technologies in following the PBL method and developing OD skills?

To achieve this, we have designed a university course on OD following the Design Based Research (DBR) methodology, which uses learning environments as teaching and learning laboratories, with the aim of making them more effective (Sandoval & Bell, 2004). DBR has been defined as “a series of approaches, with the intent of producing new theories, artifacts, and practices that account for and potentially impact learning and teaching in naturalistic settings” (Barab & Squire, 2004). By completing a DBR cycle, we were able to develop design principles that can support university educators in delivering OD education that meets students’ needs.

## Open data education

As the concept of OD is relatively new, there is only a small number of research papers that examine OD education. Some of the studies in the area provide best practices and recommendations about areas that need attention in order for the OD education to be effective, while others present case studies where OD was used in training courses (Table 1).

**Table 1:** Related work

Scope	Methods	Findings	Reference
Instructors’ motivation to use OD as learning material	Interviews/Thematic Analysis/Document Analysis	Instructors are motivated by OD characteristics Inquiry-based approach followed Need to design learning activities effectively and identify appropriate teaching tools	Coughlan (2019)
Training to promote governmental OD	Case study/Interviews/Content Analysis	Raising awareness of OD promotes their use. Training is more effective when students are familiar with the governmental context The characteristics and needs of the students need to be taken into consideration for delivering successful training	Gascó-Hernández et al. (2018)
Big Data projects for engineering courses	Case study/ Questionnaires/Expert validation	Risks/challenges/data storage technologies used were identified Standard definition of Big Data is needed Data security mechanisms and ethical code for using data are needed	Lara et al. (2020)
OD curriculum for data professionals	Feedback from webinars	Best practices for designing open educational resources	Mikroyannidis et al. (2016)
Identifying OD useful to secondary schools	Participatory design research	Datasets produced were of poor quality and scope. Data skills of the involved stakeholders were limited. Limited sustainability of the OD interventions	Selwyn et al. (2017)
Data literacy activities for children	Ethnography DBR	Students show critical thinking when involved in data collection	Wolff et al. (2019)

The literature shows that OD education is still in its infancy, thus, there is need to raise awareness of OD (Gascó-Hernández et al., 2018) and overcome risks and challenges, such as low data quality (Lara et al., 2020; Selwyn et al., 2017). Designing effective learning environments and appropriate resources is one of the priorities in the area, with scholars suggesting choosing adaptable formats, technologies and pedagogies (Coughlan, 2019; Mikroyannidis et al., 2016). However, the evidence of what works effectively in practice and in the long term is extremely limited (Selwyn et al., 2017).

In terms of pedagogy, instructors appear to prefer inquiry-based approaches (Coughlan, 2019; Lara et al., 2020). This seems to be appropriate considering that there is evidence that students’ learning is improved when they are involved in hands-on activities (Wolff et al., 2019) and become familiar with the context in which the data were created and the way real organisations operate (Gascó-Hernández et al., 2018; Mikroyannidis et al., 2016). Nonetheless, it is unclear to what extent such approaches help the students to develop OD skills (in addition to the usual ones, e.g. critical thinking, problem solving etc.), especially at a university level.

In addition, the role of technology in facilitating the development of OD skills is unclear. Lara et al. (2020) listed at least 10 different technologies used in Big Data student projects, which may be useful for engineering education, but not necessarily suitable for OD education or students from other disciplines. Besides, data analysis tools are not the only technologies used by students as part of their learning experience. The LMS used to support students’ learning, by providing access to relevant resources and opportunities to interact with the classroom, can also affect student experience.

As a response to the above gaps, we have chosen the PBL method as the theoretical lens for our enquiry, a well-established instructional approach that facilitates deep learning by motivating students to analyse real life problems (Dolmans et al., 2016). This is in line with the previous literature in OD education discussed above and will help us to explore the role that the PBL method may play in the development of OD skills. In order to design a university course that incorporates various technologies effectively, we have followed the DBR methodology, which is commonly used by studies in the field that aim to develop OD courses (Selwyn et al., 2017; Wolff et al., 2019).

## Methods

### The course

The designed OD course was elective for students in the fourth year of their undergraduate studies in Applied Informatics at a Greek university and consisted of 13 workshops that covered various topics about OD (e.g. obtaining OD, scrubbing, visualisation etc.). Initially, the learning goals were broad with the view to adjust them after the needs analysis (first step of DBR) has been completed. More specifically, at the end of the course, students were expected to:

A) Understand the usage and the importance of advanced information systems in solving business problems
B) Obtain OD and make interesting visualisations
C) Analyse OD in a way that shows their added value

The assignment of the module was designed followed the PBL method and asked students to work in small groups to solve a problem using OD. The problem was not given to the students by the instructor, but was formulated by them as part of the PBL method discussed in the next session. Initially, the team assignment counted for 100% of the students’ final mark, a decision that later was revised based on the findings of the research.

Moodle was the LMS used to support the module. Students were asked to join the platform to access the module’s learning materials (slides from the lectures, quizzes, links to OD databases etc.) and complete various tasks, such as participating in group discussions, evaluating the week’s workshop, and storing useful material. In addition, they had to use Tableau, a data visualisation software, to analyse the OD on which their problem was based.

### The problem based learning method

As the PBL method has been applied to a wide range of disciplines (from management to medicine), there is a wide range of PBL models available in the literature. While the number of steps suggested by each model varies (between 5 and 8 steps), all the models include problem analysis and plan development stages (Zotou et al., 2020). We have chosen the 9-step model developed at Aalborg University, as it is the one that has been applied in project management settings (Zotou et al., 2020), and we wanted students to treat their assignments as real OD projects. Table 2 shows the broad learning activities planned initially for the course based on the chosen model’s PBL steps.

**Table 2:** Aalborg PBL steps and the designed learning activities

PBL step	Learning activities
Group forming	Students are asked to create groups to work on the semester’s group assignment.
Problem formulation	The groups are asked to identify a problem that can be solved by showing open data’s added value. A series of lectures help the students explore OD and identify potential problems. A list of OD portals is provided to the students to support brainstorming.
Task formulation	The groups divide the problem into smaller parts and allocate tasks to the members.
Data gathering	The groups identify and retrieve suitable datasets from the recommended portals.
Analysis	The groups examine the chosen datasets and distinguish the different elements that comprise the dataset.
Design	As part of the design process of their solution, the groups are asked to create 3 interesting SPARQL queries from the RDF data they chose. Each query should provide meaningful information and have a scalable complexity.
Implementation	The groups have to create at least one interesting visualization that generates added value from the OD. In addition, they are required to write a short post within a blog, describing their work and their visualization and highlighting the added value and benefits of open data.
Evaluation	Each group is assessed weekly on their project progress, in order to avoid any misconceptions and prevent at-risk failures. At the end of the semester, the groups present their work and their findings, and receive feedback from the other groups and the instructor.
Reporting	The groups have to submit the final written report that summarises the conclusions and suggestions for future work.

As soon as the main learning PBL activities were decided, the DBR process started that allowed us at first, to identify the OD topics and learning material that should be covered during the course to support the students while completing the PBL activities and later, to tweak the course according to the students’ feedback to ensure that it remains useful to the students while they try to develop OD skills. The next section discusses the DBR process we followed and the different stages of data collection it included.

### Design-based research

**Fig. 1:**
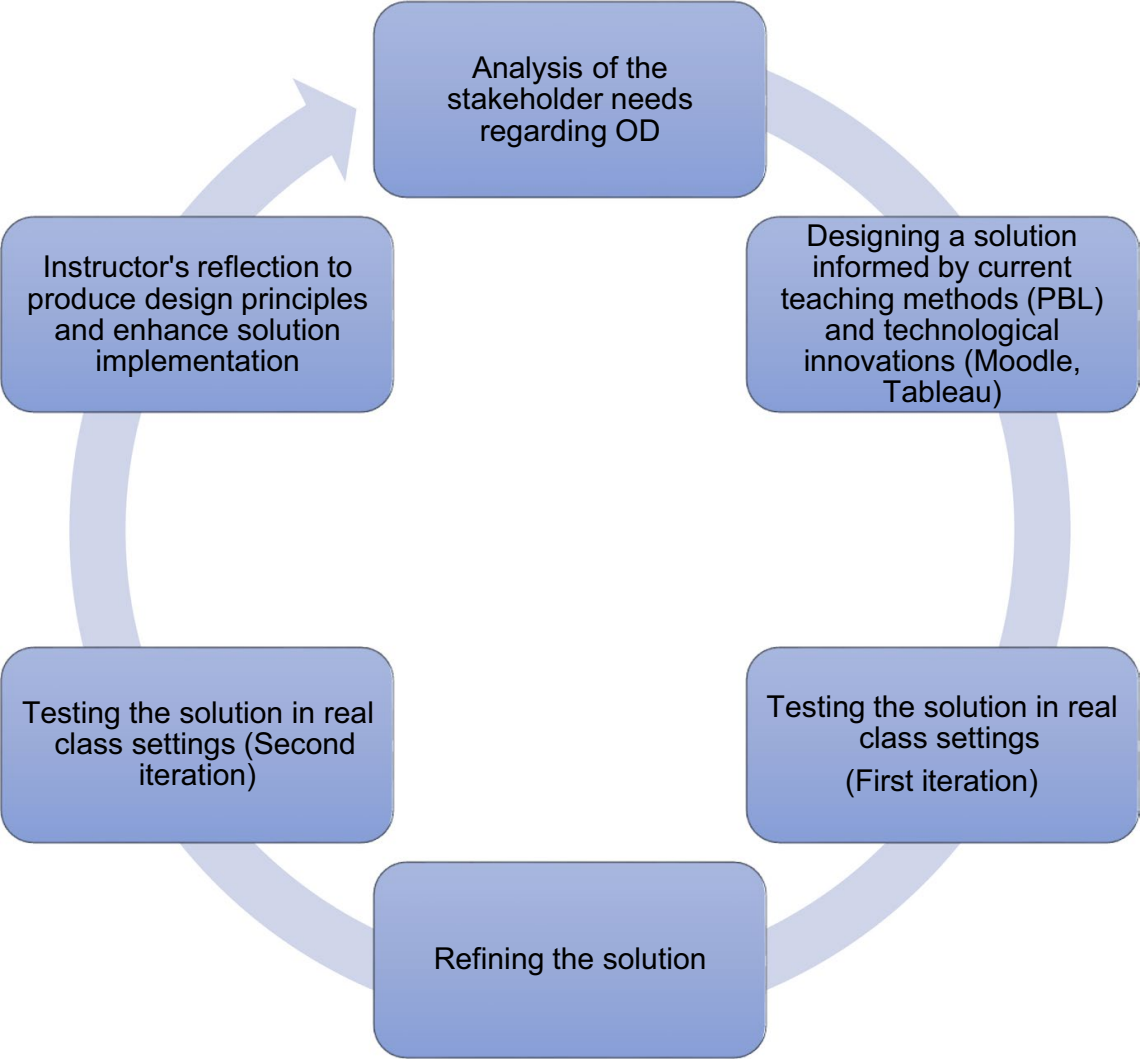
Design based research process adapted from Amiel and Reeves (2008)

Our data collection was based on a full cycle of DBR and consisted of an initial study that aimed to understand the stakeholder needs when it comes to OD education and two design iterations that allowed us to improve our teaching method and develop relevant design principles (Fig. 1).

At each DBR step, we collected data by conducting either individual interviews or focus groups with different groups of stakeholders (e.g. students attending previous deliveries of the course, students attending the first iteration of the new course etc.) (Table 3).

**Table 3:** Summary of the study’s methods

DBR step	Method	Sample	Research question
Analysis of stakeholders’ needs	Interviews	Public servants attending an OD workshop University students attending a previous OD course	OD skills
Testing the solution (first iteration)	Focus group	University students attending the 1st pilot course	PBL method and educational technologies
Testing the solution (second iteration)	Focus group	University students attending 2nd pilot course	PBL method and educational technologies
Reflection	Reflection	Instructor	All

#### Needs analysis

For the analysis of stakeholder needs, we collected data from stakeholders familiar with OD by having interviews with two groups: a) 3 public servants that were participating in the OD workgroup of a local municipality, and b) 12 university students that had attended an OD module. We chose to interview public servants due to their interest in OD. Although the number of public servants has decreased since the economic crisis, the government is still one of the major employers in Greece and its Open Data Index is at the same level with the average value for OECD countries (OECD, 2017). Thus, we expect that interviews with the chosen participants can give us a realistic view of the OD usage in Greece.

The interviews included some introductory questions that aimed to understand the participants’ experience with OD (e.g. What is your experience with OD and how did you start working with it?), questions that focused on relevant challenges (e.g. Are there things holding you back from using OD?), questions that asked for potential solutions (e.g. How do you think these issues should be addressed?), and questions about potentially useful skills (e.g. What are the most important skills when dealing with OD?). The interviews were analysed using reporting templates that were developed for the purposes of the study (see Online Resource 1) and focused on the following three areas: identified needs/problems, skills and knowledge, and target group (i.e. public sector, private sector, students or academia). The results (reported in section 4.1) allowed us to answer our first research question and identify the OD skills that would be included in the curriculum of the new course.

#### First iteration

After running the first iteration of the course, we conducted a focus group with 5 students (out of 11 that were enrolled) that volunteered to share their views about it. The main reason for choosing this type of methodology is that focus groups allow participants to discuss shared lived experiences and at the same time, they allow the researcher to explore ‘diverse understandings’, which is not usually feasible with the other research methods, such as interviews (Liamputtong, 2011, p. 5). Considering that group working was one of the main elements of the pilot, we decided that a focus group with students from each of the two assignment groups (which had 5 and 6 students respectively), would allow a more in-depth examination of their collaborative learning experience.

The focus group lasted almost an hour and the discussion started with general questions that asked the students to reflect on their whole experience with the module and identify moments when they felt they learned something new or faced difficulties with learning. More specific questions followed that aimed to start a discussion about the different elements of the learning method (e.g. learning technologies used). As it was not possible to ensure that all the students were fluent speakers of English, the focus group was conducted in the Greek language, which is the language in which the module was taught and the native language of all the individuals involved in the focus group (i.e. participants and moderators). The discussion was recorded, transcribed and analysed in Greek. Only the findings and the quotes used in the study were translated in English by the research team.

Thematic analysis, which is “the process of identifying themes in the data which capture meaning that is relevant to the research question” (Willig, 2014), was deemed as suitable for understanding the views that students had about the adopted learning method. The anonymised files with the transcriptions were read by all of the authors (Greek native speakers) to make sense of the data. The first author used a combination of coding methods: firstly, Descriptive Coding, in an attempt to generate the initial codes and secondly, Pattern Coding to identify emergent themes (Saldaña, 2013). An example of how the codes were used to develop themes can be found in the Supplementary Information section. The files of the analysis and the coder’s memos were shared with the rest of the research team using cloud storage. The codes and the themes were examined by the other authors and consensus about the names of the themes were reached after a series of group calls. The team’s meetings were also uploaded on the team’s cloud storage to create a trail of the analytical process followed.

#### Second iteration

We refined our solution based on the findings of the focus group and we tested it again in a second iteration. We conducted again a focus group with 3 undergraduate students (out of the 16 enrolled to the course) that agreed to participate in the study, following the same procedure described above. The themes derived from the two focus groups are presented in Table 5. Based on these, we were able to answer the other two research questions regarding how students view the use of the PBL method for OD education and what the role of educational technologies is in the learning experience.

#### Reflection

The final step of our design research circle was the reflection completed by the instructor (one of the authors), which was supported by learning analytics collected on Moodle (i.e. heatmaps, statistics regarding students’ activity etc.). The different data sources (i.e. stakeholders answers, students’ perceptions, and instructor’s reflection) have allowed us to develop a number of the design principles that can improve OD education in the context of our study.

## Design narrative

### Needs analysis

During the interviews, both groups of participants (i.e. students and municipal servants) agreed that their main need when it comes to OD is developing advanced skills (e.g. annotating OD, using SPARQL query language etc.). For students, acquiring basic skills (e.g. statistical analysis etc.) was equally important, which was something expected as they did not have the same working experience with public servants that felt that they already possess basic OD skills. Another area of interest was the applications of OD, with students being interested in the theoretical applications of OD (e.g. “knowledge on different kinds of applications I can create with open data”, “how to identify data that can create business value”) and public servants focusing on the applications relevant to their practice (e.g. “how to identify innovative ideas for visualizations for my city”, “how to use APIs in creating applications for the city with Open Data”). Finally, skills/knowledge related to the development of an OD culture were mentioned by both groups (e.g. “how to communicate the benefits of Open Data for entrepreneurship to citizens and public authorities”, “more information on why Open Data is important”).

When it comes to the challenges that they face regarding using OD, the municipal servants mentioned the following three:

- Difficulty to learn and develop skills that help them gather and exploit data. This is due to the fact that they are not aware of what technologies and tools are available for retrieving data from multiple sources, cleaning them so that they contain only the data that are useful, and describing them with semantic meaning. The usage of standards and existing vocabularies for creating RDF data was mentioned as an important learning area.
- Lack of knowledge of how to create dynamic and live services and applications for citizens, by exploiting multiple data sources of the city (e.g. sensors, geographical information, measurements on pollution etc.). They felt there is need to learn how to develop applications that will have intuitive and attractive dashboards and visualizations for keeping the citizens informed.
- Finding a way to encourage entrepreneurship in the city by using OD. This was considered as an important mission of the municipality due to the vast effect that the financial crisis had on the enterprises of the city. The participants felt that there is a need to understand how OD can lead to the creation of innovative start- ups and the development of services and applications that will foster economic growth.

When asked about the way of training that they would find helpful, they answered that they would prefer hands-on experience with existing OD that would allow them to learn how to use different technologies for each step of the OD circle. The ideal training should also include the creation of visualizations that would help them understand how they can later develop dashboards for the city.

The lack of essential OD skills was also deemed as a challenge for some of the students. For example, most of them considered the discovery of relatable and interesting data that can be used to create applications challenging or expressed difficulties in combining different datasets and creating visualisations based on them. The students also thought that the lack of an OD culture is also a challenge, as the concept and the applications of OD are not always clear to them. According to them, these challenges can be addressed with lab exercises and experimentation with real Open Datasets. Some of them highlighted the need for these exercises to be contextualised (e.g. case studies) as that could also help them understand the potential applications of the data.

Table 4 summarises the updated learning goals for the course, based on the skills and areas of knowledge that were mentioned during the interviews per type of participants. We addressed these learning goals in the following learning plan (parentheses indicate when students moved to the next PBL step):

1. Introduction to the course and the assignment
2. Introduction to Open Data – Open Data Applications
3. Obtaining Open Data- Open Data Refinement
4. Open Data Visualisation – Part 1
5. Open Data Analysis – Part 1
6. Group presentations (Problem and Analysis)
7. Open Data Visualisation – Part 2
8. Open Data Analysis – Part 2
9. Group presentations (Design)
10. Advanced Topics related to Open Data
11. Group presentations (Implementation)
12. Group presentations (Evaluation)
13. Conclusions (Feedback on the drafts of the projects).

**Table 4:** OD skills

Category	Skills/Knowledge that students should have gained by the end of the course	Number of participants (Public Servant: PS/ Student: S)
OD Lifecycle	Obtaining Data • Finding/ downloading data • Blending data • Converting data • Data formats	2PS 8S
	Scrubbing Data • Filtering • Quality checking • Validating/ cleaning data	1PS 3S
	Analysing Data • Statistical analysis • Creating predictions • Interpreting/ validating findings	7S
	Presenting Data • How to present data • Creating visualisations • Working with maps • Creating dashboards • Storytelling with data	3PS 6S
Culture	• What is OD? • How OD creates value • What impact OD will have • OD and open standards	3PS 4S
Academic Practice	• Role of OD in innovation/research • Publishing OD • Metadata/annotation • Legal issues/ privacy/ ethics/ security	2PS 4S
Advanced Skills	• APIs, big and Live Data • Linked data • Semantic web • Vocabularies and schema	2PS 5S

**Fig. 2:**
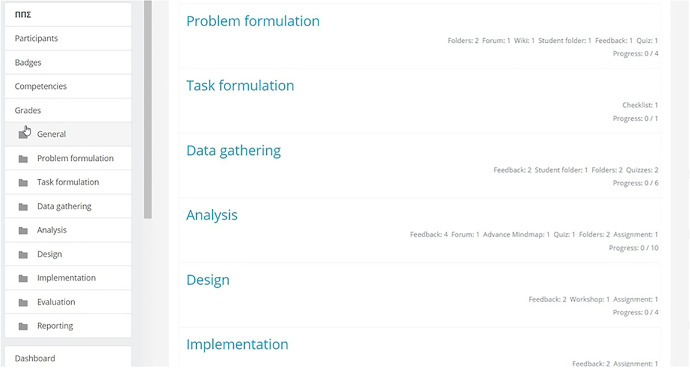
First pilot’s online environment

The online environment of the module (Fig. 2) was designed in a way that could guide the students through the PBL method. The course layout followed the topic format available on Moodle, with each of the main PBL phases (i.e. Problem formulation, Task formulation etc.) representing a separate topic.

### First iteration

The feedback we received from the first focus group shows that while students felt that they gained OD knowledge/skills, they also faced some challenges related to tutor guidance, team working, and use of the learning technologies (Table 5).

**Table 5:** Themes from the two focus groups and indicative quotes

Theme	Iteration 1	Iteration 2
Tutor guidance	Student 5: “[We needed] clear instructions about the software. Student 3: And for the assignment, regarding how it should be like… the final outcome.”	S3: Something that I liked was that in some sessions [name of one of the instructors] was there and he spent some time showing us the software. He was there in case we made any mistake and explained to us what the mistake was. I believe his presence in the seminars was quite important.
Learning outcomes	S5: We were … S1: ((quieter)) alert. S5: Yes, alert, to get involved, to work, so we learnt how to use Tableau and how we should work. […] S2: There were moments at the final stages of the assignment that I felt I learned something. R: For example? S2: When … after a lot of changes… mainly in the structure… I felt I learned about visualisations. Because we had to merge data, so we had to add new things, change the text… so in the end, we saw that that it worked and was successful. R: What about the rest? S3: We learned about Open Data, with which I was personally not familiar. R: So, on a theoretical level? S3: Yes! The module gave us new information.	S1: While working on the assignment we applied into practice everything we learned as theory in the module. You have to use the tools, so you definitely learn after this (…), you gain something, it is not just knowledge… of the software. R: So, for you, this part of the assessment was the one that made you feel that you learned; that your learning effort was successful. S2: Um, I would also say the assignment, especially when we managed to create visualisations with Tableau, that was when I said “ok, now we learned something”. S3: For me, the moment that I realised that I understand new concepts was when we had the practical seminar and I understood how all these diagrams or analyses are created […] S2: It is a valuable skill; since nowadays OD is considered valuable… and the Introduction of the module made us think that “yes, it is something good to know as apparently it is quite relevant to the outside world…” R: So, from your attendance so far, the topic of the module- S3: - I chose the module as I understood from the first sessions that although this is not a core module, it can give us basic knowledge on a whole subject, open our eyes and show us that there are many tools that we can use to our advantage.
Team working	S3: There were five of us, which helped, so if one week one couldn’t make it, there were the other four that could work, the next week the other one… There wasn’t a week that no one couldn’t make it… there was collaboration… eh… everything fine. We had split… for example, some people were responsible for the content, others for Tableau, the next week the opposite. Researcher: How do you feel about that? The fact that you are going to be assigned the same mark as a team? S2: No, it is not that fair. R: Uh- huh. S2: In general. Eh, it is fair when everyone contributes the same. S1: The ones that don’t want to work in a team, should be given the option to prepare an individual assignment.	S3: In general, I feel that I haven’t contributed at all to the assignment, although I wanted to, due to… external factors… because I was away, because I work that day before and I am too tired and I don’t have time (…). I would like to make up for this and support the team… and I think it is a little bit difficult because the team has many members and communication can be hard. R: So, there were some issues here… S1: We live far away… and the more people in the team, the harder to coordinate meetings. S2: Also, each one of us has their own schedule…There were also more people attending the module at first, so we wasted time… S1: … waiting to see who is going to enrol in the module […] R: Were there any moments you felt that you worked successfully as a team? S2: Yes, obviously… S1: When we were trying to formulate the problem, which was the hardest part. It took us so many weeks discussing about potential topics; each member did their own research and then we had group discussions to see whether we had found something.
Role of the technologies	S5: I think Moodle had some ((doubt)) unnecessary components- S3: Yes S5: That we never used. R: Any example? Something that you saw on the platform and you thought ‘what is the purpose of this?’. S2: The way that sessions were structured. R: Uh-huh S2: You were looking to find something specific and in order to find it, you had to click on a session, there was a folder with theory, you had to click on that folder, and there was a sub-folder, then there was another folder below… it was chaotic.	S2: Moodle is not user-friendly. How to explain it? The way it is structured… You click on a link and you cannot find what you are looking for… It reminds me how websites were 10 years ago… R: So, did you find it difficult to use…? S2: No, I just think the one we used last year was better. S3: Was it? Could that be because you are more familiar with it? S2: Um, this year is more clunky. S3: Well, Moodle seems to me simple, and ok, it may not be suited to my needs, but I can access it quickly.

#### Tutor guidance

Students felt that they needed more guidance by the tutor in order to complete the project. Although self-directed learning is one of the skills that students are expected to develop as part of the PBL method, a reluctance to take initiatives can be justified by their previous learning experience, which was lecture-based. In addition, the inclusion of computer-supported collaborative learning (CSCL) activities in the teaching and learning plan poses new challenges. Indeed, teacher support has been found to play a vital role in CSCL environments, as students need extra support to interpret findings and link theory to practice (Furberg, 2016).

#### Role of the technologies

Another interesting concept discussed was about the use of technology as part of the learning process. Students’ view about how technology can be utilised within the classroom was limited; the first technology that came to their mind was the Internet as a way to find information. Another interpretation that was given to the phrase “the use of technology in learning”, was including “the use of technology” (e.g. by companies) as a topic in the module’s learning plan.

Although the use of digital technologies in Greek universities is generally limited, it was still surprising that students in the area of Applied Informatics could not think of any other ways of incorporating technology in teaching. And while they expressed a positive attitude towards technology in general and described it as a necessary tool, without which they would not be able to learn about Open Data, their attitude towards Moodle was neutral at best.

It seems that in our case the chosen format for the module’s online environment confused the students instead of helping them to follow the PBL method. This may be related to the overall confusion about how the PBL method worked, mentioned above. However, even tools whose purpose was clear to the students (e.g. Forum) were not used as they found alternative ways of communication (i.e. face to face and Facebook messenger) more effective and more ‘direct’.

#### Team working

Another theme that emerged during the first focus group was group working. While some students mentioned that their experience of working with their team was positive, there were others that implied that not everyone contributed to the final project equally, thus awarding the same mark to all the team members was not fair.

#### Learning outcomes

Regarding the skills they developed, the students appeared hesitant during the first focus group. Although they referred to skills that are traditionally linked to the PBL method, such as interpersonal and group skills, problem solving, and knowledge building, they also mentioned difficulties in working with others and felt unsure about their ability to complete advanced tasks with OD. The development of other skills (i.e. time management, presentation skills, and data analytic skills) was also described by students throughout the discussion, even though these skills were not named explicitly. Theoretical knowledge was also considered as a positive outcome of the module, although some of the students expressed a preference for practical activities over theory.

### Second iteration

For the second iteration, we organised extra seminars were students got further support on how to use Tableau and added material about how the PBL method works on the platform. The feedback we got during the second focus group shows that the extra seminars were useful, as the students did not mention the lack of tutor guidance as an issue (Table 5).

We also changed the design of our online environment during the second iteration to see whether there will be any change in how students engage with it. More specifically, we organised the learning material/activities around the Open Data themes instead of using the PBL steps as a guide. However, the student experience with the platform was not improved, with students considering the online environment as not user-friendly (Table 5).

To support team working and eliminate the feeling that it is unfair for all the team members to receive the same mark while not everyone contributes equally, we introduced an individual component in the assessment (an exam that counted for 50% of the final mark; the other 50% was from the team project). However, difficulties in team working were also reported during the second focus group, with students attributing them to personal circumstances and the limited attendance of the classes (Table 5). It seems that the learning technologies did not support the communication among team members, which may be related to the platform not being perceived as user-friendly by the students.

In contrast with the first iteration, students expressed a more positive attitude towards OD and appeared more confident about the OD skills they developed (e.g. creating visualisations). This is probably linked to the additional Tableau seminars that ran during the second pilot.

### Reflection

Throughout the trials the tutors noticed that the students participated actively in all designed activities, but they preferred to execute the majority of the interactions and problem solving offline, through face-to-face meetings (which is in line with the findings from the focus groups). This made it difficult for instructors to have an overall overview of how group members worked together, how they executed each learning activity, divided their tasks and assessed their collaboration. Having said that, the Analytics Graph on Moodle showed that specific students from each team interacted with the platform, indicating that teams had assigned a leader early on.

In terms of meeting the learning outcomes, the quality of the submitted projects showed that students were able to investigate different data sources and produce visualisations that can be used to make sense of the large amounts of primary data and create significant value. For example, students were able to create a map with the frequency of children’s mortalities across the world during the 1990s or develop graphs that showed the main reasons of car accidents in Greece for a specific period of time by using OD. While this does not necessarily mean that students developed all the OD skills mentioned in Table 4, it confirms the students’ views that they developed at least basic OD skills.

## Discussion and implication

### Open data skills for university students

Our findings suggest that the OD learning plan we developed can tackle the common challenges considered by university students namely, acquiring basic OD skills and realising the potential of OD culture. More specifically, students participating in our pilots mentioned mostly basic skills (e.g. visualisations) when asked about what they felt they learned from the module. In addition, they expressed a positive attitude towards OD, which according to Gagné (1985) is one of the main types of learning.

However, it is unclear whether the students developed the more advanced skills mentioned as important by the practitioners (i.e. utilising APIs and contextualising OD). A reason for this might be that they were not familiar with the governmental context, which according to a previous study is a requirement for OD training to be effective (Gascó-Hernández et al., 2018). Focusing on one problem/case study may not suffice to solve that challenge. Thus, we recommend that the students are introduced gradually to relevant OD applications:

- Design principle 1: Use multiple (smaller) problems to introduce students to local OD applications.

### Using the PBL method in OD education

Overall, the stakeholders in our study found the PBL method suitable for learning OD, as solving problems using real OD has allowed students to realise its potential and become familiar with OD tools. Also, the PBL method allows the hands-on training with real data mentioned as the preferred training method during the Stakeholder Needs stage. In addition, students reported the development of team working skills (e.g. formulating the problem together), despite the few issues reported about disengaged students. This is encouraging, considering that Greek students are not used to working in teams, as exams is the most used type of assessment in Greek universities.

What we learned from delivering the module is that students may need additional support to understand how the PBL method works and what their role is within it. The seminars and the PBL material mitigated the students’ feeling that they are abandoned from the tutor. This is line with previous research that found that a tutor-led problem-based approach can support collaborative learning in computer science education (Wang & Hwang, 2017). Therefore, we recommend them as an essential step for OD education:

- Design principle 2: Develop/design seminars and learning materials that introduce students to the PBL method instead of focusing solely on OD.

### The role of educational technologies

While Tableau appeared to support our students in developing OD skills, Moodle did not seem to help them follow the PBL method and was not fully utilised. Moodle’s learning analytics showed that the forum was barely used, as students accessed only the “Announcements” and “Questions about the module” topics and ignored the thread “Discussion about finding data”. Based on the focus groups, that was because it was considered non user-friendly and students preferred other tools (e.g. Facebook messenger). In retrospect, the forum did not support e-mail notifications, and thus students could not be informed whenever a new post was created in the platform. Another potential reason for non-participation could be that students expected the tutor to start the discussion. Indeed, providing sentence openers and suggesting that all students check the forum a couple of times per week have been found to be effective strategies for facilitating online discussions (Ak, 2016; Chen & Huang, 2019).

However, the lack of participation was not limited to the forum. Other collaborative tools, such as Wikis and the Student Folder (intended to be used by students to exchange resources), were used for reporting (students uploaded their team’s meeting minutes) rather than collaboration. This could be explained by the small size of the class and the fact that students were able to have face-to-face meetings, which limited the need to communicate online. In such settings, the teams usually assign a representative that uses the LMS tools on behalf of the team (Zotou et al., 2020). However, even studies that focus on larger cohorts have found that students are not enthusiastic about LMS collaborative tools, such as forums and wikis (Hamutoglu et al., 2020).

This is unfortunate as effective collaborative tools could have solved the group working issues mentioned by students, by allowing students that were not able to attend the physical meetings to contribute equally to the group work. A potential solution is for the instructor to promote OD cloud-based tools (e.g. Tableau Online) for online team working over traditional LMS tools that students have to use in addition to any specialised software.

- Design principle 3: Encourage students to actively use cloud-based OD tools to support online collaboration and enable the participation of distance learners.

Apart from the collaborative aspect, students did not seem to like the structure of the learning environment either and specifically, having the learning material organised around the PBL steps, as they could not easily find what they needed. After organising the material around the OD topics, the feedback improved, but was not entirely positive. Some students referred to the platform as ‘clunky’ and ‘outdated’. This suggests that the problem was not the way the learning material was initially organised, especially since the literature suggests that student performance is greater when the online material is organised based on the learning approach and not around the content (García-Cabrero et al., 2018). Moodle’s design does not seem to be the problem either, as students expressed a favourable attitude for previous versions of the platform. It is likely that the lack of understanding of how the PBL method works, which was mentioned previously, created the confusion, along with the use of many subfolders. Thus, we expect that keeping the structure of the online material simple and introducing students to the PBL steps could be a more appropriate solution.

- Design principle 4: Organise the online material around PBL steps to guide students through the PBL method.

Finally, our students expressed a positive attitude towards using Tableau and despite the reported challenges, they felt that they learned how to use it effectively. From the instructor’s perspective, Tableau is a helpful tool for teaching OD as its user-friendly interface and available online tutorials make it ideal for beginners. While more studies are needed to confirm the effectiveness of Tableau for teaching OD, this is a first step towards identifying the appropriate tools that can support the use of OD in learning activities, which is listed as a current challenge in the literature (Coughlan, 2019).

- Design principle 5: Select established platforms designed for beginners when developing OD learning activities for university students.

## Conclusions

Our study has shown that while the PBL method can help students develop OD skills, an introduction to the PBL steps is needed if students do not have any prior experience with the method. Using multiple problems as part of the PBL method is also advised in cases where the students are not familiar with the local OD ecosystem. In addition, the potential of the available online tools in supporting the PBL method is not fully utilised. Our design principles aim to tackle the issue by providing guidance to instructors about how they can incorporate online tools successfully.

By following DBR, our study focused on the specific context in which the pilots took place, namely the Greek learning and working environment. Thus, future studies could try to test the ecological validity of our design principles by focusing on different contexts. Like with all qualitative studies, there is potential bias in the participants’ answers, as they express only their personal views about the PBL method and OD education. It is also likely that only the most engaged with the course students volunteered to participate in our study and their views may differ from the views of the students that for whatever reason (e.g. lack of time, lack of interest about the course’s topic etc.) decided to not engage with the course and/or study. Another limitation that stems from the study’s qualitative methodology is that we tested our approach in small groups. Thus, large-scale quantitative studies could help towards increasing the findings’ generalisability. Finally, our study used only Tableau to support the development of OD skills by the students. Future studies could experiment with different platforms to help identify additional teaching tools for effective OD training.

## Supplementary Information

ESM 1 (DOCX 36.4 kb)

## Data Availability

A sample of the dataset is provided as online supplementary information to this submission. The whole dataset that supports the findings of this study is available on request from the corresponding author. The data are not publicly available due to privacy or ethical restrictions.
