# Supplementary material for: Using the problem based learning method and educational technologies to teach open data: A design-based research approach
Source: Educ Inf Technol (Dordr). 2022 Mar 21;27(6):8859–82. doi: 10.1007/s10639-022-10995-9 (PMC8935114; doi:10.1007/s10639-022-10995-9)
Supplement: Supplementary file 1 — (DOCX 36.4 kb) [file 10639_2022_10995_MOESM1_ESM.docx]

**Supplementary information**

Appendix 1. List of abbreviations

| *Abbreviation* | *Description* |
| --- | --- |
| API | Application Programming Interface |
| CSCL | Computer-Supported Collaborative Learning |
| DBR | Design Based Research |
| LMS | Learning Management System |
| OD | Open Data |
| PBL | Problem Based Learning |
| RDF | Resource Description Framework |

Appendix 2. Example of reporting template used to analyse Student’s responses

| **Reporting template: needs analysis per respondent** | | | |  |
| --- | --- | --- | --- | --- |
| **Respondent number and name** | | 1. Student A | |  |
|  |  |  |  |  |
| *Instructions: please report 1 need/problem per row. There are multiple skills, knowledge and target groups possible per need/problem. For the skills and knowledge column put (S) for skills and (K) for knowledge. If there is no solution or skill, put '/'.* | | | |  |
| *There is a drop down list for the target group.* | | | |  |
| *Example down below. Delete when filling in.* | | | |  |
|  | **Needs/problems** | **Skills and knowledge** | **Target group** |  |
| 1 | Where to find open data | Knowledge of existing open data portals (local, regional, country level) (K) | Students |  |
| EXAMPLE |  | Knowledge of how datasets have been tagged with metadata (K) |  |  |
|  |  | How to search an open data portal (S) |  |  |
|  |  | How to navigate an open data portal (S) |  |  |
| 2 | How to identify the added value of open data | How to search an open data portal (S) | Students |  |
|  |  | How to combine different datasets (S) |  |  |
|  |  | Knowledge on linking open data (K) |  |  |
| 3 | How to evaluate the quality of open data | Knowledge of cleaning data (K) | Students |  |
|  |  | Knowledge of evaluation criteria for good quality of data (K) |  |  |
|  |  | How to determine the quality level of open data (S) |  |  |
| 4 | How to work with open linked data | Knowledge on retrieving open data e.g. using SPARQL (K) | Students |  |
|  |  | How to think differently and change my existing mindset on working with triplets instead of traditional relational databases (S) |  |  |
| 5 | How to exploit statistical open data | How to search for statistical open data (S) | Students |  |
| 6 |  | Knowledge on how to perform analytics on statistical data (K) |  |  |
| 7 | There are no services that allow easy visualization of open data | How to navigate to a service that allows me to view datasets (S) | Students |  |
|  |  | Knowledge on what each type of visualization's added value and purpose (K) |  |  |
| 8 | How to make profit / innovate from open data | Knowledge on existing success stories with open data (K) | Students |  |
|  |  | How to combine different datasets for added value (S) |  |  |
|  |  | How to publish my data (K) |  |  |
|  |  | Knowledge on different kinds of applications I can create with open data (K) |  |  |
| 9 | How to decide which vocabularies to use for creating RDF data | Knowledge on different vocabularies and their usage (K) | Students |  |
|  |  | How to determine which vocabularies are more useful (S) |  |  |
| 10 | How to create my own open data | Knowledge on opening up and publishing open data (K) | Students |  |
|  |  | How to determine which data would be beneficial to be opened or published (S) |  |  |

Appendix 3. Example of reporting template used to collect responses

| **Persona: Student with ICT background and programming skills** | |
| --- | --- |
| **Needs (I want/need to learn/know/find out)** | **Comments** |
| what open data are |  |
| why open up data |  |
| how open data creates value |  |
| which data are of value/needed/demanded |  |
| about open data licenses, why and how to apply those |  |
| how to filter out sensitive data | yes it is important to address legal and ethical barriers |
| what is considered sensitive / private data | yes it is important to know exactly which data is considered sensitive and require cleaning or transformation |
| how can I check the quality of data |  |
| how to clean data | yes cleaning data is important before starting their analysis |
| how to annotate data so that they are correctly understood |  |
| more about data formats |  |
| how to convert from one format to another | very important to transform data to different formats, because right now the majority of the data has been opened up in non usable formats |
| which visualization is best for the type of data/user/problem addressed |  |
| how to create a visualization | very important (one image = a thousand words) |
| how to annotate a dataset so it is easier to be found |  |
| where to find open data that could be useful for my own processes/research |  |
| how to blend open data with my own | combining different datasets and defining relations |
| how to use open data to create something |  |
| how can I make money out of my creation |  |
| how to make my creation sustainable |  |
| how to make my creation discoverable |  |
| how to collaborate with stakeholders |  |
| how to set up a business model |  |
| how to perform analytics with my open data | yes very important |
| where can I find success stories that will show me open data’s potential |  |
| how can I contribute to the entrepreneurial sector by using open data |  |
| how to link / reconcile data with other data | linking data is very important for discoverability |
| how to store and access / retrieve my annotated data |  |
| how to publish my open / linked data |  |

Appendix 4. Examples of reporting template used to collect response

| **Reporting Template: OD challenges** | |
| --- | --- |
| **Aspects** | Description |
| **Name of the organization** | Student B |
| **Sector** | Academia |
| **Give a detail description of the challenges (The challenges that they are facing regarding Open Data, what makes these challenges so important to solve and describe the constraints)** | It is very challenging to learn how to create meaningful information out of data that is available in different sources around the Web. Once you understand more about this new concept, it becomes interesting and more easy to approach. |
| **Describe the best method(s) of training (include why)** | A more suitable method would include weekly assignments, hands-on experimentation during class, and involvement with multiple different datasets from various domains (e.g. health, environment etc.) |

| **Reporting Template: OD challenges** | |
| --- | --- |
| **Aspects** | Description |
| **Name of the organization** | PS A |
| **Sector** | Public sector |
| **Give a detail description of the challenges (The challenges that they are facing regarding Open Data, what makes these challenges so important to solve and describe the constraints)** | The most prominent challenges regarding Open Data is the difficulty to learn and develop skills that help us gather and exploit this data. We are not aware of what technologies and tools are available for retrieving data from multiple sources, cleaning them so that they contain only the data that is useful and describing them with semantic meaning. The usage of standards and existing vocabularies for creating RDF data is important for us to know and gain skills on. Furthermore, an important challenge is the creation of dynamic and live services and applications for our citizens, by exploiting multiple data sources of our city (e.g. sensors, geographical information, measurements on pollution etc.). We need to learn how to develop applications that will have intuitive and attractive dashboards and visualizations for our citizens to be informed and aware. It is also our mission to encourage entrepreneurship in our city, which because of the financial crisis has deteriorated significantly. Thus, we want to see how Open Data can lead to the creation of innovative start ups and the development of services and applications that will foster economic growth. |
| **Describe the best method(s) of training (include why)** | The best training method would be a hands-on experience with existing Open Data where we can learn different technologies for each step of the Open Data circle. We want to be able to actually use the tools instead of learning about them only, so that we can also develop the skills of handling open data. This way we can later transfer our knowledge and skills to other employees and to our team. The training should also include the creation of visualizations that will help us see how we can later develop dashboards for our city. |

Appendix 5. Developing themes

| **Example of developing a theme based on identified codes from focus groups 1 (FC1) and focus group 2 (FC2)** | |
| --- | --- |
| **Name of the Theme** | Tutor guidance |
| **Codes (separated with semi-colon)** | issues with the tutor’s instructions (FC1);  need for practical sessions (FC1);  need for clearer feedback (FC1);  consistency in the project’s requirements (FC1);  frustration due to poor communication (FC1);  instructor’s support (FC2);  influence from the instructor (FC2) |
| **Description** | The theme captures the role that tutors’ guidance played on the experience of the students. |
| **Focus group excerpts** | Codes identified in the sample excerpts (translated from Greek):  **Issues with the tutor’s instructions**  S1: … But more specifically, there were some issues regarding the topic… how it was explained. So, the assignment, let’s say, was on… what we worked…it was not difficult, it’s just, the problem was that, uh, at some stages, we couldn’t understand, uh, how to work… How it should be completed… all the work, because we assumed that… uh, we need to have some knowledge on how to use some tools…  R: So, as you have already told me that it would be useful to have, uh, clearer explanation. Um, maybe some theoretical framework?  S1: Mainly practical.  R: A practical framework?  S2: Slightly more specific instructions.  R: What recommendations do you have about the course, so it can be better?  S5: [We needed] clear instructions about the software.  S3: And for the assignment, regarding how it should be like… the final outcome.”  R: OK  S3: Or, if there are no specific requirements, then it should be really up to us… so, one of the two.  S2: Because it seems that we are going to be marked based on something specific, we need to know from the beginning how to… where we need to go. Because if we weren’t marked on that specific thing, we could have developed the project as each team wanted.  **Need for practical sessions**  S1: … To search properly, that is. There was a need for a basis, a common ground, at least. To have one or two seminars, or whatever it takes, in which we would be given some basic information about how the software works.  RL: Like a tutorial?  S1: Yes, and then we could give it a try. But we needed these instructions, to be told that in this way you can do this, in that way you can do that.  **Need for clearer feedback**  R: Anything else…? And apart from the fact that there wasn’t so clear… clear instructions?  S2: [interrupts] I think that was the most important  R: OK… It was…  S1: And a little bit more… clarifying… uh… the feedback to be more…  R: More…?  S1: instructive  **Consistency in the project’s requirements**  R: How would you like to receive feedback? What would help?  S2: To… be more stable. That is, the first time, we started working, we created a 10-page Word document, and we came to the class, and we were told that [pause]: “this should be in an article format, two pages”  R: OK  S2: Which was something new… The next time…  S3: [interrupts] We brought two pages, um, - “that’s not so good actually, the previous version was better”.  R: OK  S1: It could be more … [pause] specific. So, we know…  R: So, for example, if you had a list with the requirements…  S1: Yes, and to know that the project should have, let’s say, these five specific characteristics.  R: I see.  S2: So, we had to go to the class as teams and present what we had done, and based on the instructor’s feedback, either to change the whole structure of the assignment, or change specific parts, so what the girls say, and I agree too, is that we should have been given 5-10… standards so we could work based on them.    **Frustration due to poor communication**  S1: There was a point when… no one could understand what we had to do.  R: So, there was a general…?  S1: So, again the issue was… understanding.  R: Uh huh  S1: And when three people, no one can understand what has to be done… we didn’t know what direction we should take.  R: Was there an option to use a forum?  S4 and S5: [in agreement, unsure] No…  S3: We didn’t know it existed basically.  R: OK.  S3: We were not given any…  S2: [interrupts] Forum, you mean whether we could talk via Tableau? Oh, yes, the instructor told us that we could do that.  **Instructor’s support**  S3: Something that I liked was that in some sessions [name of one of the instructors] was there and he spent some time showing us the software. He was there in case we made any mistake and explained to us what the mistake was. I believe his presence in the seminars was quite important.  **Influence from the instructor**  R: So, in general, how did you find the course?  S1: Uh, quite interesting.  R: Yes?  S1: Our understanding is that it is something that you can use after you finish your studies, it is something that you can also use for… some job, or to create something on your own, for example, an application, let’s say, that will use Open Day, or something like that.  R: So, you found it interesting generally speaking?  S2 and S1: Yes,  S2: Definitely, it is something like a skill set, as the instructor told us that the Open Data is now considered as …, it has value let’s say, and with this, with the introduction that he gave us, we thought that yes, it is something useful, and from what we did [in the course], we see that it links to the real world, it is not something irrelevant. |
| **Searching for themes** | After the first focus group, it was clear that the students faced many challenges with the semester’s project, mainly due to the lack of (clear) instructions/expectations from the instructor. The frustration they felt seems to be either about the project itself (the structure of the assignment) or the way the software (e.g. Tableau) works. We have captured this with separate codes (e.g. Need for practical sessions, Need for clearer feedback etc.), which, however, are clearly linked to each other (at the end of the day, it is about the how well students felt they were supported by the instructor). After the changes made to the course (i.e. second iteration), the students’ attitude is clearly different; there are no complaints, but rather appreciation of the support they received from the tutor during the practical sessions, and even influence in terms of how they see Open Data (students seem to adopt the instructor’s view that Open Data is valuable). Despite the codes from the two focus groups being different, there is a clear common theme that emerges i.e. the effect that the instructor’s guidance can have on students’ experience with the course, which can be either negative (if the guidance is limited) or positive (if there is clear guidance from the instructor). For this reason, the theme was named ‘tutor guidance’, which captures both the positive and negative effects. |
|  |  |
